# Mechanical Venous Thrombectomy for Deep Venous Thrombosis in Cancer Patients: A Single-Center Retrospective Study

**DOI:** 10.1007/s00270-024-03691-3

**Published:** 2024-03-28

**Authors:** Riya M. Patel, Koustav Pal, Syed Hadi Ahmed, Joshua D. Kuban, Milan Patel, Ketan Shah, Peiman Habibollahi, Zeyad Metwalli, Varshana Gurusamy, Sanjay Gupta, Cristhiam M. Rojas-Hernandez, Vahid Afshar-Kharghan, Michael H. Kroll, Rahul A. Sheth

**Affiliations:** 1 Department of Interventional Radiology, University of Texas MD Anderson Cancer Center, 1515 Holcombe Blvd., Unit 1471, Houston, TX 77030-4009 USA; 2 Section of Benign Hematology, University of Texas MD Anderson Cancer Center, Houston, TX USA

**Keywords:** Venous thromboembolism, Mechanical thrombectomy, Cancer, Endovascular

## Abstract

**Purpose:**

Venous thromboembolism (VTE) is a major contributor to the mortality of cancer patients. Mechanical thrombectomy (MT) is an endovascular technique that physically removes a thrombus without thrombolytics. The purpose of this study was to evaluate safety, efficacy, and clinical outcomes following MT for lower extremity DVT in cancer patients.

**Methods:**

This single-center, retrospective study evaluated outcomes following MT of lower extremity DVT in cancer patients from November 2019 to May 2023. The primary outcome measure was clinical success, defined as a decrease in Villalta score by at least 2 points following the intervention. Secondary outcomes included repeat intervention-free survival and overall survival. Technical success was defined as restoring venous flow with mild (< 10%) or no residual filling defect.

**Results:**

In total, 90 patients and 113 procedures were included. Technical and clinical success was achieved in 81% and 87% of procedures performed. Repeat intervention-free survival at 1 month, 3 months, and 6 months post-procedure was 92%, 82%, and 77%, respectively. The complication rate was 2.7%. Pathologic analysis of the extracted thrombus revealed tumor thrombus in 18.4% (18/98) samples. Overall survival for the study cohort was 87% at 1 month, 74% at 3 months, and 62% at 6 months. Patients who were found to have tumor thrombi were noted to have a decreased overall survival compared to patients with non-tumor thrombi (*P* = 0.012).

**Conclusion:**

MT is safe and efficacious in reducing cancer patients’ VTE-related symptoms. The high rate of tumor thrombus in thrombectomy specimens suggests this phenomenon is more common than suspected.

**Graphical Abstract:**

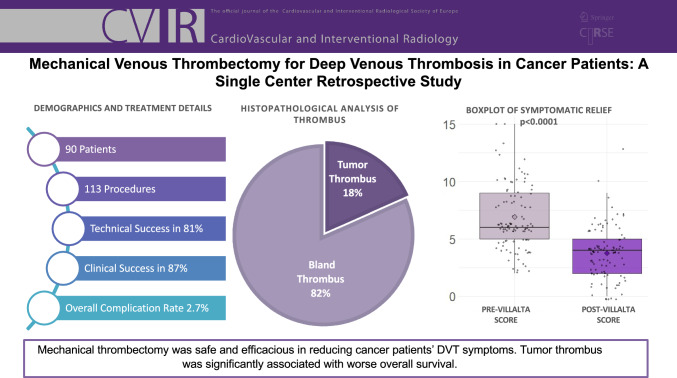

**Supplementary Information:**

The online version contains supplementary material available at 10.1007/s00270-024-03691-3.

## Introduction

Venous thromboembolic disease (VTE), including both deep vein thrombosis (DVT) and pulmonary embolism (PE), is a major contributor to the morbidity and mortality of cancer patients. The annual incidence of VTE is 1 to 3 in every 1000 adults in the general population, which is three- to sevenfold higher amongst cancer patients [1]. The increased risk of thrombosis in cancer patients can be attributed to disturbances in Virchow’s triad [2]. The observed rate of VTE in cancer patients has increased over the past two decades; this may be attributable to an increased thrombosis risk from newer anti-cancer therapies, such as tyrosine kinase inhibitors and immunotherapies [1, 3–8].

For some patients on anticoagulation, symptom relief from VTE may take an unacceptably long time. Furthermore, anticoagulation alone is known to be insufficient in preventing the chronic sequelae of cancer-associated DVT, such as post-thrombotic syndrome (PTS). The clinical manifestations of PTS negatively impact the quality of life, thus forcing the need for more effective treatment options.

As an adjunct to therapeutic anticoagulation, endovascular therapies have proved capable of restoring blood flow through thrombosed veins while yielding long-term symptom relief [9]. In the cancer patient population, there are often many reasons to avoid using thrombolytics, such as recent surgery, history of bleeding, or brain metastases. Alternatively, mechanical thrombectomy is an endovascular technique that physically removes the thrombus without thrombolytic medications. The ATTRACT trial is the largest prospective randomized trial, which evaluated catheter-directed interventions in managing VTE [10]. Although the ATTRACT trial did not demonstrate a significant reduction in the incidence of post-thrombotic syndrome in patients with deep vein thrombosis treated with catheter-directed therapies, a reduction in the severity of PTS symptoms was observed in patients with iliofemoral DVT. The standard treatment for cancer-associated VTE is anticoagulation therapy. However, the ATTRACT trial did not include cancer patients. Apart from this trial, there are limited data on the outcomes of mechanical thrombectomy procedures, especially those performed on cancer patients. The purpose of this study was to evaluate safety, efficacy, and clinical outcomes following mechanical thrombectomy for lower extremity DVT in cancer patients.

## Methods

### Ethics Statement

This single-institutional retrospective cohort study adhered to the Health Insurance Portability and Accountability Act and was approved by the institutional review board with a waiver of informed consent.

### Study Design and Participants

The institution’s database was queried for patients who underwent mechanical thrombectomy from November 2019 to May 2023. Inclusion criteria included all cancer patients with lower limb, pelvic, or inferior vena cava DVT. Patients were included irrespective of symptom duration, contraindication to thrombolytics, and prior history of DVT. Patients with upper extremity DVT and patients who underwent thrombolysis were excluded. Renal insufficiency or severe iodinated contrast allergy was not considered exclusion criteria, as the institutional practice in this clinical setting is to perform thrombectomy with intravascular ultrasound (IVUS) and fluoroscopic guidance without using iodinated contrast.

Patients were closely examined in the hospital in the days following the procedure for recurrent symptoms or complications. Follow-up information was assessed based on clinical follow-up notes or repeat imaging (US, CT, fluroscopy) wherever available.

The electronic medical record was reviewed to assess the preprocedural location of the thrombus, the extent of the thrombus, and symptom duration. Symptoms related to thrombosis were categorized as pain, swelling, or redness on the affected limb. Pre-procedural cross-sectional imaging was used to assess for potential causes of DVT, such as external compression due to tumors when applicable. The risk of venous thromboembolism in cancer patients was determined using the Khorana scoring system [11]. DVT symptom severity was quantified using the Villalta scoring system, which assesses five patient symptoms and six physician-assessed clinical signs, each on a scale of 0 to 3 for a final score between 0 and 33 [12, 13].

### Procedural Details

Mechanical venous thrombectomy (MT) was performed by 5 interventional radiologists with at least 3 years of experience in venous interventions. The majority of procedures were performed under general anesthesia, or at the discretion of the anesthesia department. The access site and device selection were at the operator’s discretion, as was the use of IVUS. All cases had the goal for complete thrombus removal. Patients were initiated on systemic anticoagulation prior to the procedure, and intraprocedural activated coagulation time (ACT) was measured to ensure adequate anticoagulation with a target of 250 s. The popliteal vein was most commonly accessed in the supine, frog-legged position. The common femoral vein was accessed for patients who did not have a thrombus involving the femoral vein. Hydrophilic wires and catheters were used to traverse the thrombosed vein, after which the access site was dilated to accommodate the mechanical thrombectomy device. Typically, aspiration catheters were employed in the presence of indwelling devices such as stents and IVC filters, while `stent-retriever’ style devices were used otherwise. Rheolytic thrombectomy devices such as Angiojet require the use of thrombolytics, and most cancer patients have at least a relative contraindication to this medication. A major advantage of mechanical thrombectomy is the lack of reliance on thrombolytics. Therefore, this approach is rarely used at the authors’ institution, a cancer center.

The decision to place a stent to alleviate extrinsic compression was determined based on residual luminal narrowing after maximal thrombus extraction.

### Definition of Outcome Parameters

The primary outcome measure was clinical success, defined as a decrease in Villalta score by at least 2 points within one day following the intervention. Secondary outcomes included repeat intervention-free survival, the time interval from the initial thrombectomy procedure, and any subsequent intervention to restore or improve lower extremity venous patency. Venography images were evaluated to characterize the degree of thrombus removal and to assess for residual stenosis from pre-procedure and post-procedure scans. Technical success was defined as restoring venous flow with mild (< 10%) or no residual filling defect. Overall survival was measured in days from the date of mechanical thrombectomy to the date of death or the last follow-up. Complications were categorized based on the Common Terminology Criteria for Adverse Events (CTCAE) classification system. Primary patency and complications were evaluated according to the recently published reporting standards document [14]. Occurrences of procedure-related complications were assessed in addition to residual filling defects on post-intervention angiography and the need for a blood transfusion. The characterization of the extracted thrombus as a tumor versus a bland thrombus was determined by the presence of tumor tissue in the thrombus using standard histologic analysis.

### Statistical Analysis

Demographic, procedural data and complications were reported using descriptive statistics. The Kaplan–Meier product-limit estimator generated survival curves and sustained patency rates. Competing risk regression was performed to evaluate the cumulative incidence of repeat intervention, with death treated as a competing risk. The paired *t*-test was performed to observe for pre- and post-procedural change in Villalta scoring. The Chi-square test was performed to observe differences in survival and repeat intervention-free survival. Chi-square tests were used to examine whether the distribution of cancer types differed between patients with tumor thrombus and those with bland thrombus. *P* < 0⋅050 was considered statistically significant in all analyses. Statistical analysis was performed with R software version 4.2.3 (R Foundation for Statistical Computing, Vienna, Austria).

## Results

### Patient Demographics and Treatment Characteristics

From November 2019 to May 2023, 90 patients with 113 procedures were included. The median follow-up time was 6 months (range: 0.2–39 months). The demographic characteristics of patients are summarized in Table 1. The median age was 58 (IQR, 51–65), with 42 female and 48 male patients. 60% of treated patients had a prior DVT, 37% had an IVC filter, and 8.9% had prior stenting. Gastrointestinal cancer was the most common underlying malignancy (*n* = 19), followed by genitourinary (*n* = 13) and squamous cell carcinoma (*n* = 13). All patients had metastatic stage IV cancer. The median symptom duration of these patients was 14 days (range 0– 303 days), and the most frequent symptoms included leg swelling, leg pain, and color change. The median Khorana score was 1 (range 0–5), while the median pre-procedure Villalta score was 6 (range 0–18). The Villalta score was not recorded for eight procedures, so these were excluded from the analysis of this outcome measure. Most patients (*n* = 78, 69.0%) experienced a unilateral DVT, with 30 patients treated for bilateral disease and five patients with a central DVT of the inferior vena cava. Of the patients with unilateral DVT, 41 were symptomatic on the left leg, and 37 were on the right leg.

**Table 1:** Patient demographics

Patient demographics	
Characteristic	*n* = 90
Age (median in years, IQR)	58 (51, 65)
Gender	
Female	42 (47%)
Male	48 (53%)
Cancer diagnosis	
Breast cancer	1 (1.1%)
CNS malignancies	3 (3.3%)
Genitourinary malignancies	13 (14%)
Gastrointestinal malignancies	19 (21%)
Gynecologic malignancies	12 (13%)
Hematologic malignancies	7 (7.8%)
Lung cancer	7 (7.8%)
Melanoma	4 (4.4%)
Sarcoma	10 (11%)
Squamous cell carcinoma	13 (14%)
Thyroid carcinoma	1 (1.1%)
Prior IVC filter placement	
no	57 (63%)
yes	33 (37%)
Prior venous stent placement	
no	82 (91%)
yes	8 (8.9%)
Prior DVT	
no	36 (40%)
yes	54 (60%)

### Technical Variates

The procedural characteristics are shown in Table 2. In most procedures, a mechanical thrombectomy was performed by a ClotTriever catheter (*n* = 79) or FlowTriever catheter (*n* = 45) (Inari Medical, Irvine, CA, USA). AngioJet (Boston Scientific, Natick, MA, USA) and Penumbra (Irvine, CA, USA) were other devices used. Angioplasty alone was performed in 61%, whereas angioplasty combined with stenting was performed in 39% of procedures. Blood transfusion was performed in 18 procedures (16.0%) to treat chronic anemia or replace blood losses using the FlowTriever device. The distribution of thrombus is presented in Table 3. Post-procedural anticoagulation was administered after 113 procedures as follows: low molecular weight heparin in 60 (53.1%), unfractionated heparin infusion in 28 (24.8%), Xa inhibitors in 22 (19.5%), direct thrombin inhibitors in 2 (1.7%), and none in 1 (0.9%).

**Table 2:** Procedural details

Procedural detail	
Characteristic	*n* = 113
Laterality	
Bilateral	30 (27%)
Central	5 (4.4%)
Unilateral	78 (69%)
Among unilateral	*n* = 78
Right	37 (47.5%)
Left	41 (52.5%)
Symptom duration (median in weeks, IQR)	2 (1, 4)
Location	*n* = 113^b^
Femoropopliteal	44 (39%)
Iliofemoral	79 (70%)
Iliocaval	61 (54%)
IVC	32 (28%)
External compression	
yes	54 (48%)
Khorana score at first presentation of DVT^a^	*n* = 89^a^
0	32 (25%)
1	29 (33%)
2	22 (25%)
3	17 (15%)
4	2 (2.2%)
5	1 (1.1%)
Villalta score on presentation	
0–4	20 (18%)
5–9	65 (58%)
10–14	17 (15%)
> = 15	3 (2.7%)
Not applicable	8 (7.1%)
Preoperative anticoagulation	
Direct thrombin inhibitor (Bivalirudin)	1 (0.9%)
Unfractionated Heparin Infusion	33 (29%)
LMWH	40 (35%)
none	12 (11%)
Xa inhibitor	27 (24%)
Perioperative anticoagulation	
Unfractionated Heparin Infusion	43 (50.2%)
LMWH	33 (38%)
Xa inhibitor	10 (12%)
Thrombectomy device	
ClotTriever	79 (70%)
FlowTriever	45 (40%)
Angiojet	2 (1.8%)
Other device	1 (0.9%)
Angioplasty with stenting	
No	69 (61%)
Yes	44 (39%)
Complications	
No	107 (95%)
Yes	3 (2.65%)

^a^Khorana score was calculated at the time of first DVT

^b^Thrombi may be located in more than one site per procedure

**Table 3:** Outcomes following mechanical thrombectomy

Outcome	*n*^a^	*n* = 113
Symptomatic relief following thrombectomy	111	
No		22 (19%)
Yes		89 (79%)
Thrombus histology	98	
Bland thrombus		80 (82%)
Tumor thrombus		18 (18%)
Presence of residual stenosis	113	
None		91 (81%)
Partial		17 (15%)
Complete		5 (4.4%)
Use of IVUS	111	44 (40%)
Blood transfusion given	113	18 (16%)
Post-procedure anticoagulation	113	
Direct Thrombin Inhibitor		2 (1.7%)
Unfractionated Heparin Infusion		28 (24.8%)
LMWH		60 (53.1%)
No postoperative anticoagulation		1 (0.9%)
Xa Inhibitor		22 (19.5%)

^a^Values unknown were excluded, lowering *n* for these variates

### Outcome Measures

#### Technical Success

For the entire study cohort, technical success was achieved in 80.5% (91/113) of procedures performed.

#### Clinical Success

**Fig. 1:**
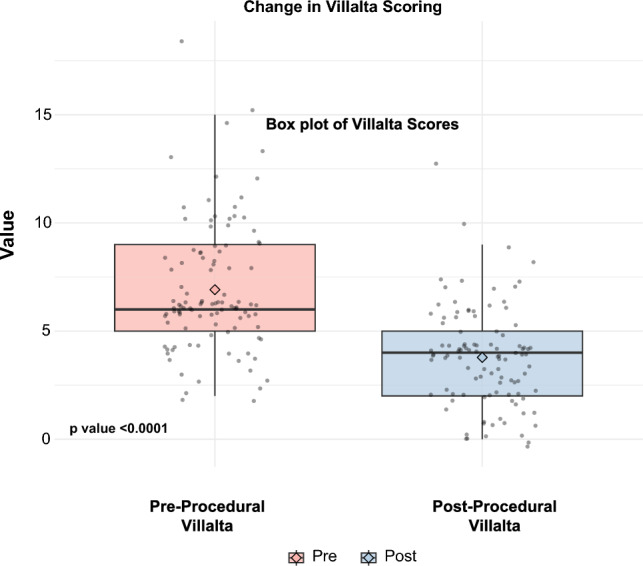
Box plot demonstrating Villalta scores prior to and following mechanical thrombectomy. There was a significant decrease in Villalta scores following the intervention

For patients who had pre-procedural and post-procedural Villalta scoring (*n* = 105), clinical success was achieved in 87.6% (92 patients). Pre-procedure and post-procedure Villalta scores showed a significant improvement in symptoms, with a p-value < 0.0001, as seen through a paired *t*-test analysis (Fig. 1). The median pre-procedure Villalta score was 6, which later decreased to 4 post-procedurally. Of 113 procedures, 55 (52.3%) resulted in a Villalta score reduction of three or more points. IVUS examination was used in 44 procedures to delineate thrombus burden, with 15% of procedures having partial residual filling defects and 4.4% having complete residual filling defects. Six patients underwent mechanical thrombectomy under IVUS and fluoroscopic imaging alone without using iodinated contrast due to renal failure; all six interventions were technically successful without the need for repeat intervention.

### Repeat Interventions

**Fig. 2:**
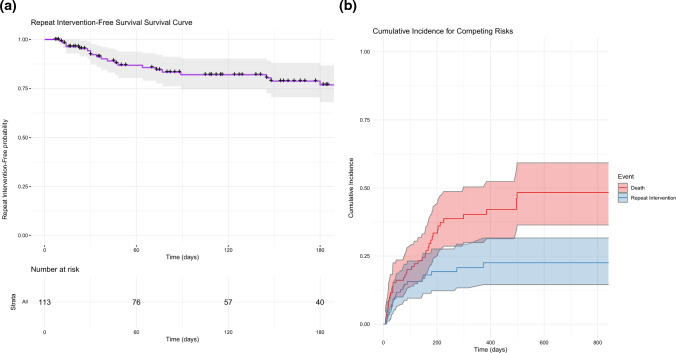
**A** Repeat Intervention-free survival. **B** Competing risk analysis for repeat intervention, with death considered as a competing risk

Repeat intervention-free survival at 1 month, 3 months, and 6 months post-procedure was 92%, 82%, and 77%, respectively (Fig. 2A). Recurrence of lower extremity DVT and repeat intervention was noted in 19% of cases (*n* = 22). One patient required two procedures for DVT in left limb and the right limb within a 2 month time frame. This was not included as a re-intervention.

### Survival and Long-Term Outcomes

**Fig. 3:**
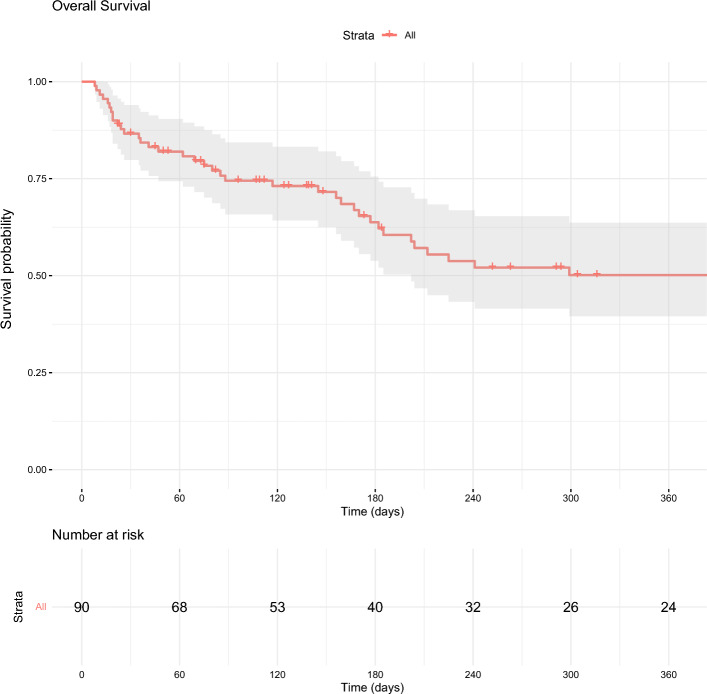
Overall survival of the study cohort

The overall survival for the study cohort was 87% at 1 month, 74% at 3 months, and 62% at 6 months (Fig. 3). Long-term outcomes at 12 months included 71% repeat intervention-free survival and 48% overall survival for the entire cohort. When stratified by thrombus histology, patients with bland thrombus had an overall survival of 51% at 12 months, while the overall survival for patients with tumor thrombus was 26%.

### Complications

The overall procedural complication rate was 2.7% (3/113). The profile of post-procedural complications included postoperative hypoxia, respiratory concerns, hemorrhage, and patient confusion upon awakening from anesthesia. The three procedures with complications were mild (CTCAE < 3) and resolved with conservative management.

### Histopathological Analysis

**Fig. 4:**
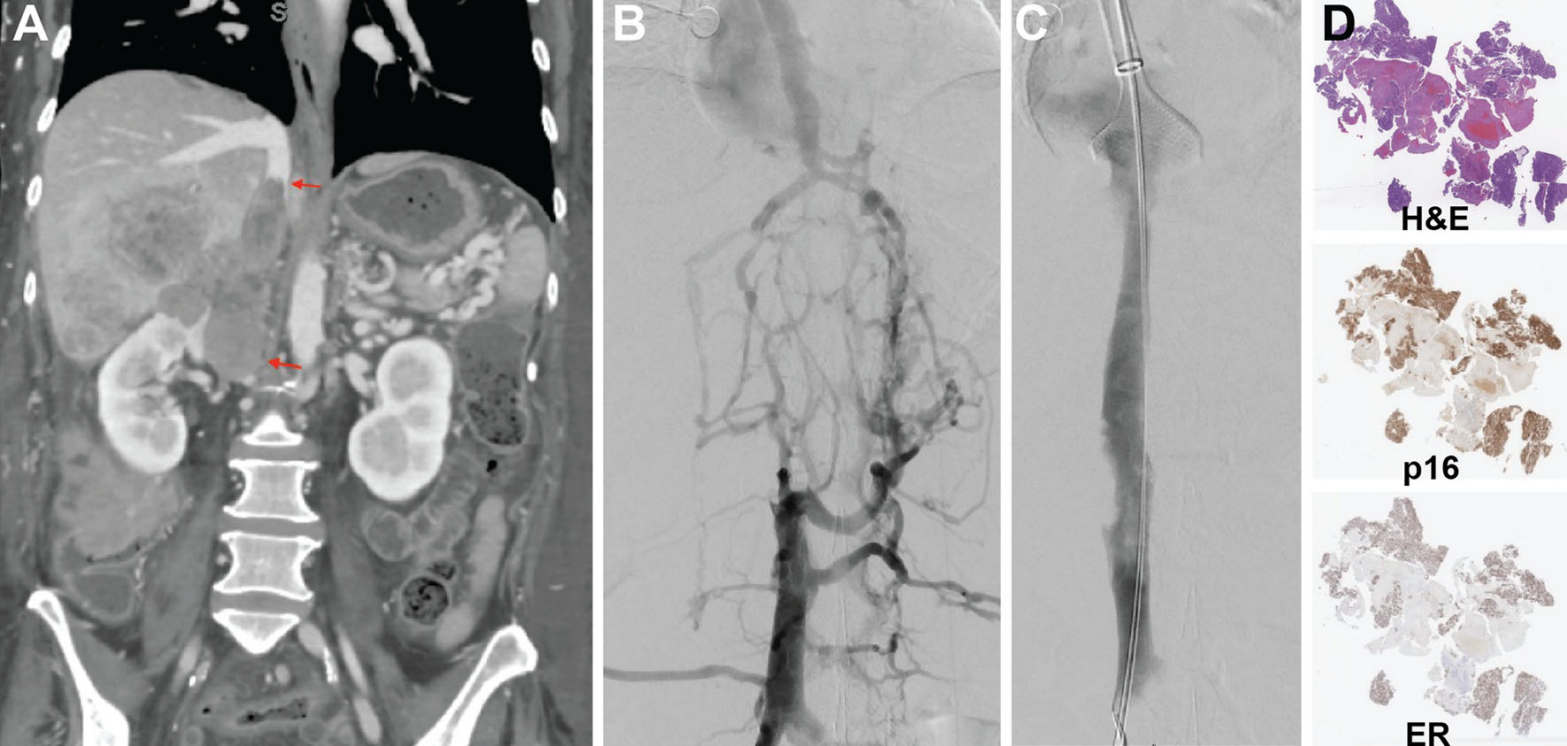
Example of patient with tumor thrombus. **A** A patient with endometrioid adenocarcinoma with 6 months of progressive lower extremity swelling and heaviness precluding ambulation was found to have a large suprarenal and intrahepatic IVC tumor thrombus. **B** Pre-intervention angiography confirmed a hemodynamically significant obstruction of these IVC segments. **C** Following thrombectomy, there was near complete removal of the tumor thrombus, with restoration of brisk in-line flow through the IVC. **D** Histologic analysis of the extracted thrombus demonstrated ER+ and p16+ tumor cells, consistent with the patient’s primary malignancy

**Fig. 5:**
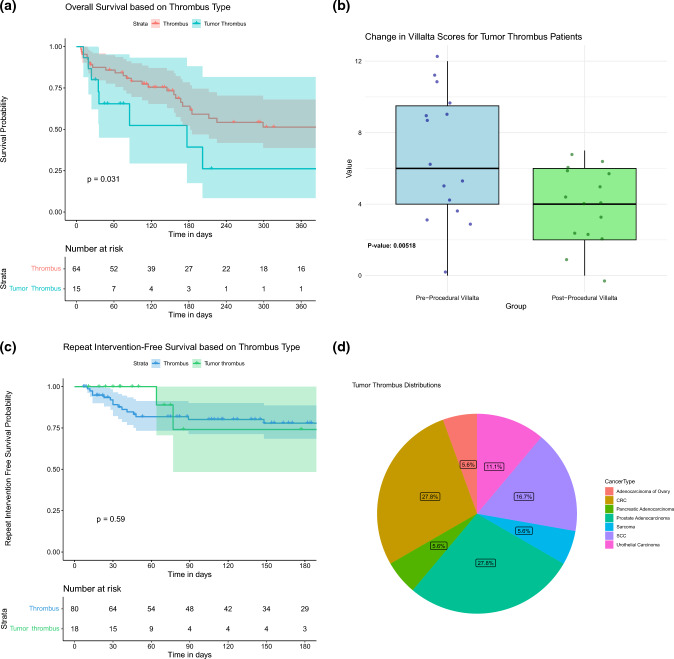
Outcomes for patients with bland versus tumor thrombus. **A** Patients with tumor thrombus on histology were found to have a significantly higher mortality than those with bland thrombus. **B** Villalta scores prior to and following mechanical thrombectomy for patients with tumor thrombus. There was no significant difference in treatment response following mechanical thrombectomy for patients with bland versus tumor thrombus. **C** Patients with tumor thrombus were not at an increased risk of repeat intervention. **D** Relative proportions of underlying histologies in patients found to have tumor thrombus. There was a significant difference in the relative proportions of cancer types among patients with bland versus tumor thrombus. **A** Overall survival stratified by thrombus histology. **B** Box plot of tumor thrombi with change in villalta scores. **C** Repeat intervention-free survival in tumor thrombus patients. **D** Relative proportions of underlying histologies in patients found to have tumor thrombus

Pathologic analysis of the extracted thrombus revealed that the thrombus was comprised of tumor in 18.4% (18/98) of samples (Fig. 4). Sixteen procedures did not have tumor samples sent to pathology. Patients who were found to have tumor thrombi were noted to have a decreased overall survival compared to patients with non-tumor thrombi (*P* = 0.012). The median OS for patients having tumor thrombus was 85 days (95% CI 35 days—upper limit not reached) (Fig. 5A). Nevertheless, tumor thrombi patients still demonstrated improvements in their DVT symptoms following mechanical thrombectomy (*P* = 0.005) (Fig. 5B). The presence of tumor thrombi was not associated with an increased risk of repeat thrombectomy interventions (Fig. 5C). There was a significant difference in the underlying malignancies represented in the patients found to have tumor thrombus compared to bland thrombus (*P* = 0.03). For example, the proportion of patients with prostate cancer (28% vs. 2.5%), colorectal cancer (28% vs. 10%), and urothelial cancer (11% vs. 4%) were greater in the tumor thrombus group compared to bland thrombus group; conversely, sarcoma (5% vs. 15%) was less common in the tumor thrombus group.

### Statistical Analysis

In univariate competing risks regression analysis for repeat intervention-free survival (Fig. 5A, Table 4), all evaluated variables failed to reach statistical significance, with p-values exceeding the threshold of 0.05. However, in the context of predicting repeat interventions at the 0.1 significance level, there were two variables: external compression (HR 2.25, 95% CI (0.906, 5.582), *P* = 0.081), and use of the Clotriever device (HR 0.481, 95% CI (0.21, 1.1021), *P* = 0.083).

**Table 4:** Univariate competing risks regression for repeat mechanical thrombectomy

Hazard ratios, 95% confidence intervals, and *P*-values (univariate)				
Variable	Hazard ratios	Lower CI	Upper CI	*P* value
Age	0.987	0.956	1.019	0.43
External compression	2.249	0.906	5.582	0.081
Initial Khorana score	0.573	0.291	1.129	0.11
Use of IVUS	1.191	0.507	2.794	0.69
ClotTriever	0.481	0.21	1.101	0.083
FlowTriever	1.56	0.676	3.601	0.3
Angioplasty	1.914	0.643	5.7	0.24
Stenting	1.216	0.517	2.857	0.65
HPE: tumor thrombus	0.596	0.142	2.504	0.48

## Discussion

The current study observed significant symptom relief in cancer patients treated with mechanical thrombectomy. Clinical success in this study was defined using the Villalta score; though imperfect, this metric was selected given its use in the gold standard ATTRACT study. Importantly, thrombectomy was found to be safe without major bleeding complications. However, 16% in our patient cohort did require blood transfusions. This contrasts with thrombolysis studies such as the ATTRACT trial, which reported a non-trivial incidence of major bleeding within a time frame of 10 days in the pharmacomechanical–thrombolysis group, highlighting the potential risks of thrombolytics. In contrast, our patient cohort did not have bleeding following the procedure.

Venous interventions in cancer patients require specific attention, given their often multi-factorial reasons for increased VTE risk [2]. In this study, recurrent venous thrombosis requiring repeat intervention occurred in 22 cases (19%). After the initial procedure, 22 procedures (19%) had residual filling defects. As a comparison, the CaVenT study found 15% of patients who had undergone adjunctive catheter-directed thrombolysis had recurrent VTE at the 5-year follow-up time point [15].

Likewise, the OS in our study cohort was substantially shorter than other catheter-directed interventional studies for DVT. However, in the appropriate clinical setting, a limited life expectancy should not be considered an absolute contraindication to an intervention that can improve the patient’s quality of life. The immediate improvement in Villalta scoring post-procedurally for the overall cohort (< 0.0001) and tumor subgroups (*P* < 0.001) emphasizes that palliative interventions with an appropriate risk-to-benefit profile can improve patients’ quality of life. Given the low complication profile and symptomatic relief of mechanical thrombectomy, this study may support using this procedure in appropriately selected patients.

One unexpected finding from this analysis was the relatively high rate of tumor thrombus as identified by histologic assessment of the extracted thrombus material. Rates of `bland’ versus tumor thrombus in cancer patients are not well-defined in the literature. The relatively high rate of tumor thrombus has important implications. First, this study found that the presence of tumor thrombus was correlated with a decreased overall survival. Patients with tumor thrombi were susceptible to early mortality, with more than 50% dying within 85 days of the procedure. Second, it is unlikely that anticoagulation alone is sufficient, or even effective, therapy when treating tumor thrombus; additional considerations, such as changes in systemic therapy or local interventions to cytoreduce the thrombus, including thrombectomy, may be warranted. In a study conducted by Agarwal et al., 50 patients with tumor thrombus were examined, 10 of whom were treated with only anticoagulation therapy. Interestingly, only 20% showed symptomatic improvement, while 40% experienced major bleeding, raising questions about the efficacy of relying solely on anticoagulation to manage the complications associated with tumor thrombus [16, 17].

Intriguingly, there were significant differences in the underlying histologies for patients with tumor thrombus versus bland thrombus. Though the numbers are too small to draw strong conclusions, one can speculate that tumor thrombus may be under-diagnosed based on imaging or clinical presentation alone and that for certain histologies, the suspicion for tumor thrombus should be higher.

Overall, technical success was achieved in 81% of procedures. Of the procedures which did not achieve technical success (19%, *n* = 14), the majority still reported clinical relief (10/14). However, within this subgroup, some patients had an initial Villalta score within 0–4, which is classified as having no PTS, leading to a potential drawback regarding the use of Villalta score for patients having DVT; for such patients, demonstrating treatment efficacy is not possible. Other causes of lack of clinical success included the presence of tumor thrombus. Three patients required repeat interventions, following which symptom relief was achieved.

The limitations of the current study include recall and selection bias inherent in the single-center retrospective nature of the study. The limited sample size limits the generalizability of the study findings. Furthermore, the subjectivity and reliability of the scoring systems used to assess symptom relief have been questioned [18]. A specific quality of life questionnaire was also not used, leading to further limitation in assessment. Villalta scoring does not consider symptom duration and could have underlying discrepancies from patient to patient. Follow-up Villalta scores were available for only 6 months, so the durability of symptom relief is not well evaluated in the present study. On the other hand, given the mortality rate of the study cohort, symptom palliation is, more often than not, the priority in this patient population rather than long-term post-thrombotic syndrome risk reduction.

## Conclusion

Mechanical thrombectomy is safe and efficacious for the treatment of DVT in cancer patients, with significant improvement in symptom and low rate of repeat interventions, contributing to enhanced quality of life. There is a non-trivial incidence of tumor thrombus in this patient population that correlates with poorer outcomes in this cohort, highlighting the need for future mechanistic investigations.

### Supplementary Information

Below is the link to the electronic supplementary material.

Supplementary file1 (PDF 293 KB)

## Abbreviations

ACT: Intraprocedural Activated Coagulation Time
CTCAE: Common Terminology Criteria for Adverse Events
DVT: Deep Venous Thrombosis
IVC: Inferior Vena Cava
IVUS: Intravascular Ultrasound
MT: Mechanical Thrombectomy
OS: Overall Survival
PE: Pulmonary Embolism
PTS: Post-Thrombotic Syndrome
VTE: Venous Thromboembolism
