# Supplementary material for: Mechanical Venous Thrombectomy for Deep Venous Thrombosis in Cancer Patients: A Single-Center Retrospective Study
Source: Cardiovasc Intervent Radiol. 2024 Mar 28;47(5):556–66. doi: 10.1007/s00270-024-03691-3 (PMC11074016; doi:10.1007/s00270-024-03691-3)
Supplement: Supplementary file 1 — Supplementary file1 (PDF 293 KB) [file 270_2024_3691_MOESM1_ESM.pdf]

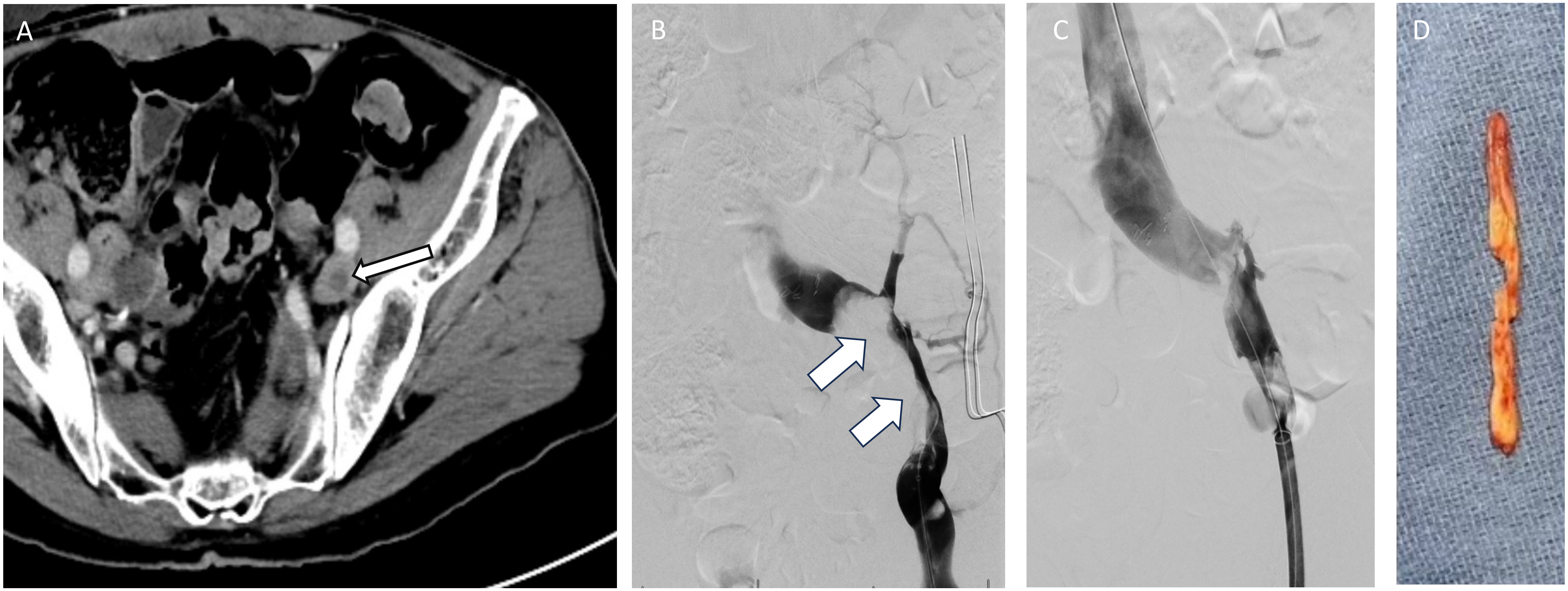

Supplemental Figure: Patient Example 2

A. A CT with contrast of the abdomen demonstrating evident tumor thrombus(White Arrow) within the left external iliac vein in a patient having urothelial carcinoma.
B. Digital subtraction venography demonstrates occlusive thrombus within the left external iliac vein.
C. Post-procedural mechanical thrombectomy, the digital subtraction venography demonstrates near complete resolution of thrombus within the left external iliac vein.
D. Photograph of the extracted tumor thrombus.
